# Long-term survivorship of an exchangeable-neck hip prosthesis with a Ti-alloy/Ti-alloy neck–stem junction

**DOI:** 10.1007/s00402-022-04634-8

**Published:** 2022-09-30

**Authors:** Massimiliano Baleani, Aldo Toni, Cristina Ancarani, Susanna Stea, Barbara Bordini

**Affiliations:** 1 IRCCS Istituto Ortopedico Rizzoli, Laboratorio di Tecnologia Medica, Via di Barbiano, 1/10, 40136 Bologna, Italy; 2 Casa Di Cura Madre Fortunata Toniolo, Bologna, Italy

**Keywords:** Hip prosthesis, Ti-alloy, Ti-alloy exchangeable-neck, Survival rate, Exchangeable-neck breakage, Fretting corrosion, Adverse local tissue reaction, Adverse reaction to metal debris

## Abstract

**Introduction:**

Breakage of exchangeable-neck (EN) and adverse local tissue reactions (ALTRs) to neck–stem junction (NSJ) damage products are responsible for increasing the revision rate of EN hip prostheses. We investigated the survivorship of an EN hip prosthesis including a NSJ with both components made of titanium alloy (Ti-alloy/Ti-alloy) to assess whether, and to what extent, EN breakage and NSJ damage affected implant survivorship.

**Materials and methods:**

Using data from a hip replacement registry, we determined survivorship of 2857 EN prostheses. Long-offset configurations of head and EN were implanted in heavy (> 90 kg) patients only in 23 hips. We investigated under which conditions EN breakages or ALTRs occurred. We also measured titanium (Ti) and vanadium (V) blood concentrations in 24 patients with a unilateral well-working prosthesis.

**Results:**

The 17-year survival rates for any reason and aseptic loosening of any component were 88.9% (95%CI 87.5–90.1; 857 hips at risk) and 96.9% (95%CI 96.0–97.6), respectively. There were two cases of EN breakage and one case of ALTR (metallosis), due to rim-neck impingement, out of 276 revisions. After an average period of 9.8 years (range 7.8–12.8 years), the maximum Ti and V blood concentrations in patients with a well-working prosthesis were 5.0 µg/l and 0.16 µg/l, respectively.

**Conclusion:**

The present incidence of EN breakage or ALTR is lower than those reported in other studies evaluating EN hip prosthesis survivorship. This study suggests that (i) the risk of EN breakage is reduced by limiting the use of long-offset configurations in heavy patients and (ii) Ti-alloy/Ti-alloy NSJ damage products do not promote ALTR nor significantly alter the rate of implant loosening. Since design decisions and implant configuration determine the NSJ strength, the NSJ strength in working conditions must be thoroughly investigated to proper define the clinical indications for any EN design.

**Supplementary Information:**

The online version contains supplementary material available at 10.1007/s00402-022-04634-8.

## Introduction

Exchangeable-neck (EN) prosthesis appeared in the second half of the 1990s. Initial clinical results were encouraging and did not reveal any role of the EN in the implant failure [1]. However, following widespread use of EN designs, including different material combinations, an increasing number of implant failures related to EN were reported. EN designs have a higher revision rate than monoblock designs in the medium term [2, 3]. As a consequence, at least four EN prosthetic designs have been recalled in the last decade and there is a growing debate on the advisability of their continued use in hip arthroplasty [2–4].

Two notable drawbacks of the EN design have a proven impact on clinical outcomes. The main drawback is the potential for mechanical failure of the neck–stem junction (NSJ), hereinafter referred to as EN breakage. This drawback is described in detail in several case reports and is included among general complications in some patient cohorts [4–6]. Another drawback is the potential biological impact of released damage products (fretting-corrosion debris and metal ions) both at local and systemic levels [7–9].

Design decisions and implant selection highly affect the risk of experiencing these drawbacks, including the NSJ alloy combination and load magnitudes (bending moments) acting on the NSJ [3, 5, 10]. Presumably, the risk of these two main drawbacks can be limited by selecting a NSJ with both components made of titanium alloy (Ti-alloy/Ti-alloy) and avoiding critical configurations leading to high bending moments, i.e., extremely long EN design implanted in heavy-weight patients.

We investigated the 17-year survivorship of a specific EN hip prosthesis, with a Ti-alloy/Ti-alloy NSJ, to test the aforementioned presumption and assess (i) whether, and to what extent, EN breakage impacted on implant survivorship, (ii) under which conditions EN breakages occurred and (iii) whether Ti-alloy/Ti-alloy NSJ damage promoted adverse local tissue reactions (ALTRs), changing the picture of implant survivorship, or affected ion concentration levels in patients with a unilateral well-working prosthesis.

## Patients and methods

### Registry study

We retrospectively reviewed a consecutive series of patients who underwent a total hip arthroplasty with an uncemented AncaFit hip prosthesis (Supplementary material, Fig. 1). All patients operated between January 2000 and December 2009—the date from when the AncaFit hip prosthesis was no longer used in the Emilia-Romagna region because it was discontinued—were identified in the regional Register of the Orthopaedic Prosthetic Implants (Supplementary material, Note 1).

The study included all patients who met all the criteria listed in Table 1. We accessed registry data to gather the detailed list of revised prosthetic components in all cases of partial or full revision. We accessed patients’ clinical records to investigate thoroughly the reason that led to revision and/or acquire complete information about the revision procedure. For cases where the reason for revision was unclear or missing, we contacted the implant manufacturer to verify whether there were incident reports (Supplementary material, Note 2) associated with those cases.

**Table 1:** Patient selection criteria

Inclusion criteria	Reason
No limits on patient demographics except for the residence: patient must live inside the Emilia-Romagna region	All patients living outside the region were excluded to avoid bias due to loss to follow-up. In fact, any surgery involving Emilia-Romagna inhabitants, even when performed outside the region, are always reported to the region itself, and thus captured by the registry
Primary total hip replacements	Secondary hip replacements were excluded due to potential bias in clinical outcomes from revision procedure of one or more components
All indications for hip replacement	No limits to avoid limiting the generalization of the results
Complete AncaFit hip prosthesis, i.e., all implant, and only those, with the subject stem and cup identified by product code	Hybrid implants were excluded due to potential bias in clinical outcomes from association of components of different prostheses
All available articulation couples, i.e., metal-on-polyethylene, ceramic-on-polyethylene, ceramic-on-ceramic	No limits to avoid limiting the generalization of the results
All available head size except for small head size: implant with 22 mm femoral head were excluded	22 mm femoral head were excluded to avoid any potential bias in the risk of dislocation

### Patient population and implant configurations

A total of 2634 patients met inclusion criteria, including 223 who also underwent contralateral total hip arthroplasty with an AncaFit hip prosthesis. Patient demographics and clinical information are listed in Table 2.

**Table 2:** Characteristics of the 2634 patients of the cohort

	Male (%)	Female (%)	Total cohort (%)
Patient demographics			
Gender	41.0	59.0	100
Age at implantation (year)			
< 40	3.4	1.1	2.0
40–49	8.5	7.1	7.7
50–59	18.6	21.8	20.5
60–69	41.0	40.3	40.6
70–79	25.7	25.5	25.6
≥ 80	2.8	4.2	3.6
Weight (kg)			
< 70	10.2	47.6	32.3
70–79	27.4	25.9	26.5
80–89	31.2	9.2	18.2
90–99	13.8	4.0	8.0
100–109	5.7	0.9	2.9
≥ 110	1.6	0.2	0.8
Missing^a^	10.1	12.2	11.3
BMI (kg/m^2^)			
Underweight < 19	0.2	1.2	0.8
Normal 19–24.9	27.8	39.2	34.5
Overweight 25–29.9	44.9	32.8	37.8
Obese > = 30	14.3	10.4	12.0
Missing	12.8	16.4	14.9
Indication for total hip arthroplasty			
Primary osteoarthritis	71.3	57.3	63.0
Femoral neck fracture sequelae	11.9	11.9	11.9
Sequelae of developmental dysplasia	8.7	24.0	17.7
Idiopathic femoral head necrosis	5.7	4.0	4.7
Rheumatic arthritis	1.0	2.0	1.6
Other	1.4	0.8	1.1

^a^This group did not include any critical head-neck configurations, i.e., patient body weight was known for all 120 critical head-neck configurations

Hip joint bearing couples for the 2857 implants included metal-on-polyethylene (6.3%), ceramic-on-polyethylene (22.1%) and ceramic-on-ceramic (71.6%) bearings. Two head diameters (28 mm and 32 mm), two EN lengths (short and long), and 12 EN designs were used in the identified patient cohort. The maximum distance of the head center with respect to the neck–stem engagement level was 40.5 mm (Supplementary material, Fig. 1). The worst-case configurations, in terms of loading condition within the NSJ, were those that maximized the lever arm and produced off-axis loading of the head center with respect to the neck–stem engagement level. We assumed all cases, where it was not possible to identify the EN orientation, as worst-case configurations, i.e., the EN oriented in the varus/retro direction. Based on this assumption, the worst-case configurations were used in 120 of the 2857 implants. However, worst-case configurations were implanted in heavy patients (body weight > 90 kg) only in 23 of the 2857 hips.

### Ion concentration in blood of patients with a unilateral, well-working, prosthesis

To investigate metal ion release in vivo, over a 24-month period we enrolled patients with a unilateral, well-functioning, AncaFit hip prosthesis who presented for routine follow-up at our Institution. Once a month, we randomly enrolled a patient among those who met all the criteria listed in Table 3.

**Table 3:** Patient selection criteria (study of the blood metal ion levels)

Inclusion criteria	Reason
Well-functioning, ceramic-on-ceramic bearing, AncaFit hip prosthesis	Minimize the bias due to metal ion release from bearing surfaces and head-neck junction
No contralateral hip prosthesis or other metal implants	Avoid bias due to other sources of metal ions
Normal range of motion of the operated hip	Impingement-free hip motion to minimize the bias due to component wear/damage
Ability to walk, sitting/rising and navigate stairs	Normal activities of daily living determine mechanical loading of the hip joint and, therefore, of the neck–stem junction
Lack of radiographic signs of loosening, i.e., radiolucent lines absent or limited to Gruen zones 1 and 7 without stem subsidence and absence of radiolucent lines or osteolysis in all the three DeLee and Charnley zones with the presence of radial trabeculae (spot welds) in zone I or II	Minimize the bias due to metal ion release from loosened components
No exposition to potential sources of metal elements (e.g., drinking well water, taking multimineral supplement, smoking, living near an industrial area, etc.)	Avoid bias due to external sources of metal ions

Blood samples (10 ml) were collected into tubes containing anticoagulant (368,381 Vacutainer, BD, Franklin Lakes, NJ), and kept frozen at − 20 °C until analysis. Titanium (Ti) ion concentration was determined using suitably prepared blood samples [11] and inductively coupled plasma optical emission spectrometry (ICP-OES, Optima 5300, Perkin-Elmer, Waltham, MA). Vanadium (V) ion concentration was determined using suitably prepared blood samples [12] and inductively coupled plasma mass spectrometry (ICP-MS, ELAN DRC II, Perkin-Elmer, Waltham, MA) equipped with dynamic cell reaction. Both procedures involved the analysis of certified reference materials as internal controls.

### Statistical analysis

The survival analysis, with 95% confidence interval (95%CI), was performed using Kaplan–Meier analysis with revision of at least one component for any cause as the endpoint. The survival times of unrevised implants were censored at the last observation, i.e., patient death or 31 December 2019, whichever came first. Statistical analyses were performed using SPSS (v14.0.1 SPSS Inc., Chicago, IL) and JMP (v12.0.1, SAS Institute Inc., Cary, NC).

## Results

### Implant survivorship and reasons for revision

**Fig. 1:**
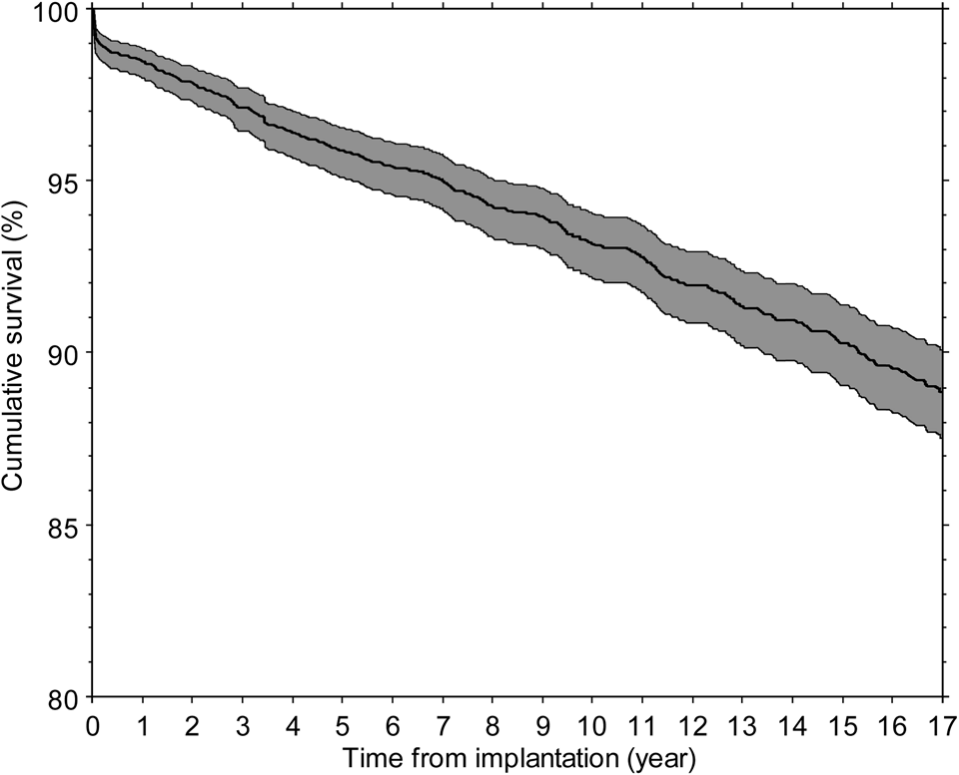
Kaplan–Meier survival curve, with 95% confidence interval

The mean follow-up, calculated including patient mortality or implant revisions, was 13.8 years (range 0.1–20.0), with a minimum of 10 years for all patients who were still alive on 31 December 2019. 17-year survival rate rates for any reason and aseptic loosening of any component were 88.9% (95%CI 87.5–90.1; 857 hips at risk, Fig. 1) and 96.9% (95%CI 96.0–97.6), respectively. Full details about the number of hips at risk and survival rate for any reason at different follow-up periods up to 17 years are reported in Table 4 (raw data are available as Supplementary material).

**Table 4:** Survival rate at different time from implantation

Time from implantation (year)	1	3	5	7	10	13	15	17
Survival rate (%)	98.5	97.2	95.9	95.1	93.2	91.3	90.3	88.9
CI	98.0–98.9	96.5–97.8	95.1–96.6	94.2–95.8	92.2–94.1	90.2–92.4	89.1–91.4	87.5–90.1
Number at risk (hip number)	2781	2690	2581	2470	2263	1942	1612	857

There were 276 revisions (Table 5), 63 of which involved revision of the cup/head only, and 213 involved partial or full revision of the EN stem. In 64 of those 213 cases, the femoral stem was left in place and only the EN was replaced. Among these revisions, one case of metallosis and two cases of EN breakage are relevant to the topic of this study (highlighted in bold in Table 5). We established the following details through access to clinical records: (i) the case of metallosis, described as “extensive black staining in the periprosthetic tissue” due to rim-neck impingement and generation of metallic debris, required the revision of the cup/head and EN. Following revision, the patient had a regular post-operative course with no further problems on the operated hip; (ii) one case of EN breakage occurred in a light body weight (class 70–79 kg), eighth decade, male patient, and required revision of the whole femoral stem. The prosthesis configuration included a long-straight EN and a short (− 4 mm) 32 mm head. Although the patient showed gait abnormalities, he denied any fall or trauma prior to EN breakage; (iii) one case of EN breakage occurred in a heavy body weight (class 90–99 kg), seventh decade, male patient, and required revision of the whole femoral stem. The prosthesis configuration involved a long-straight EN and a long (+ 4 mm) 32 mm head. There were no events worthy of mentioning prior to EN breakage. For the sake of completeness, stem revision was performed in both cases of EN breakage since it was impossible to disengage the distal taper of the EN from the stem. Transfemoral approach was performed in both cases and uncemented long-stem prostheses with longitudinal fins were implanted to achieve distal fixation.

**Table 5:** Reasons for revision of the 276 failed implants

Reason for revision	Cases *N*	Proportion (%)
Aseptic loosening^a^	68	24.64
Periprosthetic bone fracture	64	23.19
Dislocation^b^	44	15.94
Breakage of ceramic head^c^	31	11.23
Wear of polyethylene liner	10	3.62
Breakage of ceramic liner	8	2.90
Infection	8	2.90
Pain without loosening	7	2.54
Breakage of exchangeable-neck (EN breakage)	**2**	**0.72**
Metallosis	**1**	**0.36**
Other	5	1.81
Missing^d^	28	10.15
Total	276	100.00

^a^Aseptic loosening includes (i) cup loosening, (ii) stem loosening and (iii) whole implant loosening

^b^All dislocations occurred in hips with a 28 mm femoral head

^c^All cases involved a 28 mm Biolox® Forte femoral head: 27 short-neck, 2 medium-neck and 2 long-neck heads

^d^Despite details about these 28 revisions were not found, breakage of a component (exchangeable-neck, ceramic head or ceramic liner) of the prosthesis as reason for revision can be definitively ruled out (see text)

In addition, the seven cases of pain without loosening were closely evaluated. Evidence of a pseudotumor was not observed in any case.

Finally, there were 28 cases where the reason for revision was unclear or missing. In six out of 28 cases revision was limited to the prosthetic joint (cup/head). In the remaining 22 cases the EN and the stem were also revised. Incident reports were not issued for any of these 28 cases. Therefore, breakage of any implant component was excluded as cause for revision. Furthermore, we established that in 26 out of 28 cases patient had no further problems with the revised hip prosthesis. Of the remaining two cases, one patient had to undergo a second revision due to infection occurred 8 years after the first revision, and one patient had to undergo a second revision due to breakage of the ceramic head occurred 10 years after the first revision.

### Ion concentration in blood of patients with a unilateral, well-working, prosthesis

After an average in vivo duration of 9.8 years (range 7.8–12.8 years), median blood Ti and V ion levels in 24 patients with a unilateral, well-functioning, EN hip prosthesis were 2.8 µg/l (range 1.2–5.0 µg/l) and 0.09 µg/l (range 0.02–0.16 µg/l), respectively.

## Discussion

There is a growing debate on the advisability of the continued use of hip prostheses with an EN in total hip arthroplasty. EN breakage or biological reactions to NSJ damage products have proven to affect implant survivorship. Indeed, at least four EN prosthetic designs have been recalled in the last decade. However, EN prosthetic designs are still available in the market. Data on long-term survivorship of a hip prosthesis, with a design including a Ti-alloy/Ti-alloy NSJ, in a large case series have not been reported in the literature. Considering the serious complications that can occur with the use of EN hip prostheses, analysis of the RIPO data provides crucial context for understanding infrequent events such as EN breakage or ALTRs. In addition, this study provides insights into the risk of adverse reactions associated with the use of Ti-alloy/Ti-alloy junctions.

We investigated the long-term survivorship in a large case series of hip prostheses having a Ti-alloy/Ti-alloy NSJ. The implant survivorship is comparable to those reported for the most common monolithic designs from other registers, both at 10 [13] and 15 years [14]. The most concerning complications were EN breakage and ALTRs. The present incidence of EN breakage is lower than those reported in the other two studies describing the complications occurred in a wide patient cohort with a Ti-alloy/Ti-alloy EN prosthesis: 68 EN breakages occurred among approximately 5000 implants (1.4%) after an average time in vivo of 24 months [5]; 19 EN breakages occurred among the 2489 implants (0.76%) with a Ti-alloy neck after an average time in vivo of 8 years [15]. Higher rates of EN breakages have also been reported for the Profemur EN prosthesis (Supplementary material, Note 3) in smaller patient cohorts [4, 6]. Additionally, some EN breakages have been reported in patients with an AncaFit or a GPS—design very similar to the AncaFit stem [16, 17].

Differences in patient body weight, head and EN configuration and NSJ design determined the different rates of EN breakage observed in the different patient cohorts. Indeed, the force magnitude acting on the femoral head increases with patient body weight. Once the force acting on the femoral head is set, the head and EN configuration determines bending moments acting on the NSJ, while the NSJ design determines the amplitude of relative micromotion at the neck–stem interface [18, 19]. By increasing the micromotion amplitude, the risk of crack nucleation at the neck–stem interface increases as well [20], therefore increasing the risk of EN breakage. Since the NSJ design was the same in the present cohort, the other parameters (force magnitude and head and EN configuration) need consideration. We found that worst-case configurations of head and EN were implanted in heavy patients only in 23 cases. This means that, even neglecting the case where a potential bias due to gait abnormality cannot be excluded, we found one case of EN breakage out of 23 “critical” hips (4.3%), an occurrence that is anything but rare. Therefore, increasing the bending moments acting on the NSJ (through use of long EN configurations with large-diameter heads implanted in heavy patients) increases the risk of EN breakage, as already suggested by other authors [5, 10]. Consistent with this theory, EN breakages described in the literature occurred in patients with high body mass index implanted with worst-case configurations, i.e., always including long exchangeable necks and often assembled with large-diameter heads (Supplementary material, Table 1). All these figures support the theory that the low incidence of EN breakage occurred in our patient cohort was correlated with the limited use of long-offset stems in heavy-weight patients. However, even when the incidence is low, EN breakage remains an important drawback for EN hip prostheses. Indeed, stem revision may be extremely challenging due to lack of proximal attachment sites, risk of femoral fracture and diminished bone stock for fixation of the new implant.

It could be argued that, even when EN breakage does not occur, Ti-alloy/Ti-alloy NSJs release ions or damage products promoting adverse local and systemic biological responses. Metal ions can originate from the Ti-alloy/Ti-alloy NSJ, as demonstrated through our analysis of metal ion concentration in blood. Indeed, the present data fall in the upper end, or even exceed, the reference values for Ti and V ions, i.e., 0.1–1.5 µg/l and 0.01–0.09 µg/l respectively. The present data are also in agreement with blood Ti (mean 2.0 µg/l, range: 1.4–3.6 µg/l) and V (upper 95th percentile of the distribution: 0.30 µg/l) ion concentration measured by Barry et al. [21] and Catalani et al. [12] in patients having a well-functioning prosthesis with a Ti-alloy EN. Metal shell surface, shell-liner junction, head-neck junction, EN surface, NSJ or stem surface are all potential sources of Ti and V ions. Therefore, it is not possible to determine to which extent the NSJ contributes to ion release in vivo. However, the present Ti ion concentration exceeds the range of blood Ti ion concentration (0.6–1.0 µg/l) measured at 5 years for well-working monolithic stem, i.e., Ti-alloy stem including a 36 mm Co-alloy head and crosslinked polyethylene liner [22]. Therefore, a contribution of the Ti-alloy/Ti-alloy NSJ to blood ion concentration must be acknowledged even in well-working EN hip prostheses, although no significant differences were found in serum Ti ion concentration between EN and monolithic stem design [23]. In addition to metal ions, damage products can also originate from the Ti-alloy/Ti-alloy NSJ [18]. These damage products may promote ALTR. However, ALTR similar to those described in short/medium-term studies involving mixed-metal NSJs (at least one component made of Co-alloy) [9, 24, 25], have never been reported in the clinical records of this patient cohort. The only case of tissue reaction (metallosis) was likely related neck–rim impingement. Indeed, the stem was left in place at revision surgery and no further clinical problems were reported following the replacement of the cup/head and exchangeable-neck. In the literature there are no reports describing adverse local immune-mediated tissue reactions with an all-Ti-alloy EN hip prosthesis. EN implants associated with an ALTR involving lymphocytes have included at least one component made of Co-alloy [26]. Even when the titanium components were addressed as the source of particulate determining the tissue reaction, the Ti-alloy/Ti-alloy NSJ appeared “pristine” [27] and, therefore, it was not likely involved in promoting the tissue response.

Ti-containing debris might also play a subtle role in the pathogenesis of implant loosening as high concentrations of Ti-debris induce adverse response of synovial fibroblasts, osteoblasts and macrophages [28]. However, this concern does not seem to be substantiated by the present implant survivorship, using revision of any component for aseptic loosening as the end point. Even assuming that all 28 cases, where the reason for revision was unclear or missing, had to be included in the aseptic loosening group, the 17-year survival rate for aseptic loosening would only decrease to 95.5% (CI 94.5–96.3).

Despite the low occurrence of EN breakages and ALTRs, the present survival rates are lower than those reported for some monolithic designs [29, 30]. In the present study 276 revisions were reported, which is about 10% of the overall number of implants. In addition to aseptic loosening, periprosthetic bone fracture, dislocation and breakage of ceramic head mainly determined the present implant survivorship and deserve brief comments.

Breakages of ceramic head always involved 28 mm heads made of Biolox^®^ Forte ceramic. This drawback is known, as previous reports have shown that overall incidence of breakage of ceramic head in patient cohort with Biolox^®^ forte heads falls in the range 0.2–1.4%, with the 28 mm short-neck head showing higher fracture rate [31, 32].

Periprosthetic bone fracture is one of the main reasons for revision reported also in other patient cohorts implanted with an AncaFit stem [33] and was the main factor that contributed to stop the use of the AncaFit stem in Australia [34]. It seems that the shape of the AncaFit stem may lead to some degree of overload (hoop stresses) in the proximal femoral cortex and/or promote a cantilever effect of the stem tip on the lateral femoral cortex increasing the risk of femoral fracture.

Dislocation is a complication also for EN designs [1, 2, 6], even if these designs should indeed reduce the risk of dislocation. In this study, all revisions for dislocation involved a 28 mm femoral head. Although we acknowledge that the risk of dislocation decreases by increasing the head diameter [35], the aforementioned advantage is still to be proven.

We acknowledge certain strengths and limitations in this study. The two major strengths are: (i) we had no case lost to follow-up for the 2857 hips. Indeed, we included in the study only patients living inside the Emilia-Romagna region to ensure the capture of any surgery involving hip prostheses or patient death; (ii) all cases where breakage of a component of the implant occurred were identified. Indeed, even in 28 out of 276 revisions the reason for revision was unclear or missing, we were able to definitively rule out the breakage of an implant component. On the other hand, this study has some limitations: (i) revision surgery was considered as a definition of prosthesis failure. Therefore, we had no information about the functional outcomes after hip arthroplasty; (ii) no data about pre- and post-operative leg length, frontal offset and neck anteversion were available. Therefore, we could not gather any evidence about the effectiveness of using EN designs for restoring the hip center of rotation, which, however, was beyond the scope of this study; (iii) we investigated the clinical outcomes of a hip prosthesis with a Ti-alloy/Ti-alloy NSJ. Considering the difference between Ti- and Co-alloy, both in terms of mechanical behavior and potential biological impact of released damage products, any comparison with complications reported in the literature must exclude reports where the combination of different alloys for the NSJ was included or where the alloy of these two components was not reported; (iv) we measured Ti and V blood concentrations in a sample of patients with a unilateral well-functioning prosthesis. Therefore, these data were not representative of any ongoing failure of the prosthesis; (v) we had 28 out of 276 revisions where the reason for revision was unclear or missing. Although we could rule out the breakage of an implant component, these 28 cases—if the reason for revision were known—would change the percentage of the other reasons for revision but would not alter the whole figure of implant survivorship.

## Conclusions

The survivorship of the uncemented AncaFit hip prosthesis, an all-Ti-alloy EN hip prosthesis, was lower, or even comparable, to those reported for the most common monolithic designs from other registers. The present incidence of EN breakage or ALTR was lower than those reported in other studies evaluating EN hip prosthesis survivorship. It is confirmed that EN breakage is a potential drawback of a Ti-alloy/Ti-alloy EN hip prosthesis. However, limited use of long-offset configurations, with femoral heads up to 32 mm in diameter, in heavy (> 90 kg) patients mitigates the risk of occurrence of EN breakage in the present study. Under these conditions Ti-alloy/Ti-alloy NSJ damage does not promote ALTR, nor significantly impacts on the risk of occurrence of implant loosening. Although the performance of a Ti-alloy/Ti-alloy NSJ is specific to each design, and must be deeply investigated in preclinical studies, surgeons who opt to use an EN hip prosthesis must be aware that there are limitations to the clinical use of Ti-alloy/Ti-alloy NSJs.

## Supplementary Information

Below is the link to the electronic supplementary material.

Supplementary file1 (PDF 58 kb)

Supplementary file2 (PDF 75 kb)

Supplementary file3 The AncaFit acetabular component was a Ti-alloy (Ti6Al4V) porous acetabular shell assembled with a polyethylene or ceramic liner. The femoral component was an anatomically shaped stem, made of Ti-alloy (Ti6Al4V), with an exchangeable-neck, made of Ti-alloy (Ti6Al4V). The exchangeable-neck stem had a proximal 12/14 taper allowing the assembly of a Co-alloy or ceramic head. Two head diameters (28 mm, 32 mm) and three head lengths (S, M, L) were available in the inventory. Additionally, two neck lengths (short and long neck) were available. Therefore, the distance of the head center with respect to the neck–stem engagement level ranged from 22.0 mm to 40.5 mm. Six neck versions were available: straight, 8° and 15° angled neck in antero/retro direction, 8° angled neck in varus/valgus, and two hybrid neck designs combining 4° angle in varus/valgus and 6° angle in antero/retroversion (not shown in the scheme to make the figure clearer). Note, the 15° angled neck in varus/valgus was not available at that time (TIF 45916 kb)

Supplementary file4 (TXT 6 kb)

## Data Availability

Raw data have been uploaded as electronic supplementary material.
