# Supplementary material for: Long-term survivorship of an exchangeable-neck hip prosthesis with a Ti-alloy/Ti-alloy neck–stem junction
Source: Arch Orthop Trauma Surg. 2022 Sep 30;143(6):3649–57. doi: 10.1007/s00402-022-04634-8 (PMC10192172; doi:10.1007/s00402-022-04634-8)
Supplement: Supplementary file 1 — Supplementary file1 (PDF 58 kb) [file 402_2022_4634_MOESM1_ESM.pdf]

SUPPLEMENTARY MATERIALS: NOTES

Note 1

RIPO has a capture rate of about 95% of all procedures involving hip prostheses undertaken in all orthopedic departments, both public and private, located in Emilia-Romagna region. Additionally, RIPO captures any hip replacement or revision involving Emilia-Romagna inhabitants, even when performed outside the region. The design of this register was conceived to allow comparison with the most important national registers. (RIPO website https://ripo.cineca.it/authzssl/index.htm).

Note 2

By the Italian law, surgeons are obliged to immediately report any incident involving a medical device, including the breakage of any component of a joint prosthesis, to the Italian Ministry of Health and to the implant manufacturer (Italian Legislative Decrees no. 46/1997 and no. 37/2010). Failure to report an incident results in a significant financial penalty for the surgeon. Additionally, the surgeon is liable for up to 6 months detention. Therefore, the odds that a surgeon does not report an incident involving a medical device are extremely low.

Note 3

Profemur was the name of a group of stems with different shapes but having, at least until 2009, the same Ti-alloy/Ti-alloy neck-stem junction of the AncaFit investigated in the present study. Some reports have not been cited in the text because they also included Co-alloy exchangeable-necks, which have been made available in the inventory of the Profemur implant starting from 2009 (FDA 510K Premarket notification no. K091423).
