# Supplementary material for: Long-term survivorship of an exchangeable-neck hip prosthesis with a Ti-alloy/Ti-alloy neck–stem junction
Source: Arch Orthop Trauma Surg. 2022 Sep 30;143(6):3649–57. doi: 10.1007/s00402-022-04634-8 (PMC10192172; doi:10.1007/s00402-022-04634-8)
Supplement: Supplementary file 2 — Supplementary file2 (PDF 75 kb) [file 402_2022_4634_MOESM2_ESM.pdf]

Table 1 Case reports published on modular neck fractures (NS = Not Specified)

Reference	Age (year)	Femoral stem	Neck size and orientation	Head size (mm) and length	Body weight (kg)	BMI (kg/m2)
[1]	30	Profemur Z	Long, straight	NS, NS	110	29
[2]	66	Profemur Z	Long, retroverted	NS, NS	127	NS
[3]	55	Profemur Z	Long, varus	NS, NS	113	31
	67	Profemur Z	Long, varus	NS, NS	100	29
[4]	62	Profemur Z	Long, retroverted	32, long	84	26
[5]	49	Profemur Z	Long, varus anteverted	56, short	154	39
[6]	59	Profemur Z	Long, AR/V1	52, long	91	29
[7]	51	Profemur Z	NA	48, NS	NS	39
[8]	53	Profemur	Long, varus	50, NS	NS	28
[9]	42	GSP	Long, straight	28, long	110	30
	54	Profemur Z	Long, varus	28, medium	95	33
	57	Profemur Z	Long, varus	28, medium	100	35
	37	Profemur Z	Long, straight	28, medium	107	34
	56	GSP	Long, straight	28, long	110	32
	51	Profemur Z	Long, straight	36, medium	80	26
[10]	60	Profemur Z	Long, varus	28, short	88	28
[11]	38 [a]	Ancafit	Long, straight	28, long	NS	32
		Ancafit	Long, straight	28, long		

[a] The patient underwent contralateral total hip arthroplasty one year after the first surgery.

Reference

1. Atwood SA, Patten EW, Bozic KJ, et al (2010) Corrosion-induced fracture of a double-modular hip prosthesis: A case report. J Bone Joint Surg Am 92:1522–1525. https://doi.org/10.2106/JBJS.I.00980
2. Dangles CJ, Altstetter CJ (2010) Failure of the modular neck in a total hip arthroplasty. J Arthroplasty 25:1169e5-1169e7. https://doi.org/10.1016/j.arth.2009.07.015
3. Skendzel JG, Blaha JD, Urquhart AG (2011) Total hip arthroplasty modular neck failure. J Arthroplasty 26:338e1-338e4. https://doi.org/10.1016/j.arth.2010.03.011
4. Wilson DAJ, Dunbar MJ, Amirault JD, Farhat Z (2010) Early failure of a modular femoral neck total hip arthroplasty component: A case report. J Bone Joint Surg Am 92:1514–1517. https://doi.org/10.2106/JBJS.I.01107
5. Wright CG, Sporer S, Urban R, Jacobs J (2010) Fracture of a modular femoral neck after total hip arthroplasty. J Bone Joint Surg Am 92:1518–1521. https://doi.org/10.2106/JBJS.I.01033
6. Ellman MB, Levine BR (2013) Fracture of the modular femoral neck component in total hip arthroplasty. J Arthroplasty 28:196e1-196e5. https://doi.org/10.1016/j.arth.2011.05.024
7. Silverton CD, Jacobs JJ, Devitt JW, Cooper HJ (2014) Midterm results of a femoral stem with a modular neck design: clinical outcomes and metal ion aNSlysis. J Arthroplasty 29:1768-1773. https://doi.org/10.1016/j.arth.2014.04.039
8. HerNSndez A, Gargallo-Margarit A, Barro V, et al (2015) Fracture of the modular neck in total hip arthroplasty. Case Rep Orthop 2015:591509. https://doi.org/10.1155/2015/591509
9. Fokter SK, Rudolf R, Moličnik A (2016) Titanium alloy femoral neck fracture-clinical and metallurgical aNSlysis in 6 cases. Acta Orthop 87:197–202. https://doi.org/10.3109/17453674.2015.1047289
10. Zajc J, Predan J, Gubeljak N, et al (2019) Modular femoral neck failure after revision of a total hip arthroplasty: a finite element aNSlysis. Eur J Orthop Surg Traumatol 29:717–723. https://doi.org/10.1007/s00590-018-2314-8
11. Fokter SK, Gubeljak N, Predan J, et al (2021) Bilateral neck fracture in bimodular femoral stem after primary total hip arthroplasty: a case report. BMC Musculoskelet Disord 22:1–7. https://doi.org/10.1186/s12891-021-04210-y
