# Supplementary figures and images for: Long-term survivorship of an exchangeable-neck hip prosthesis with a Ti-alloy/Ti-alloy neck–stem junction
Source: Arch Orthop Trauma Surg. 2022 Sep 30;143(6):3649–57. doi: 10.1007/s00402-022-04634-8 (PMC10192172; doi:10.1007/s00402-022-04634-8)

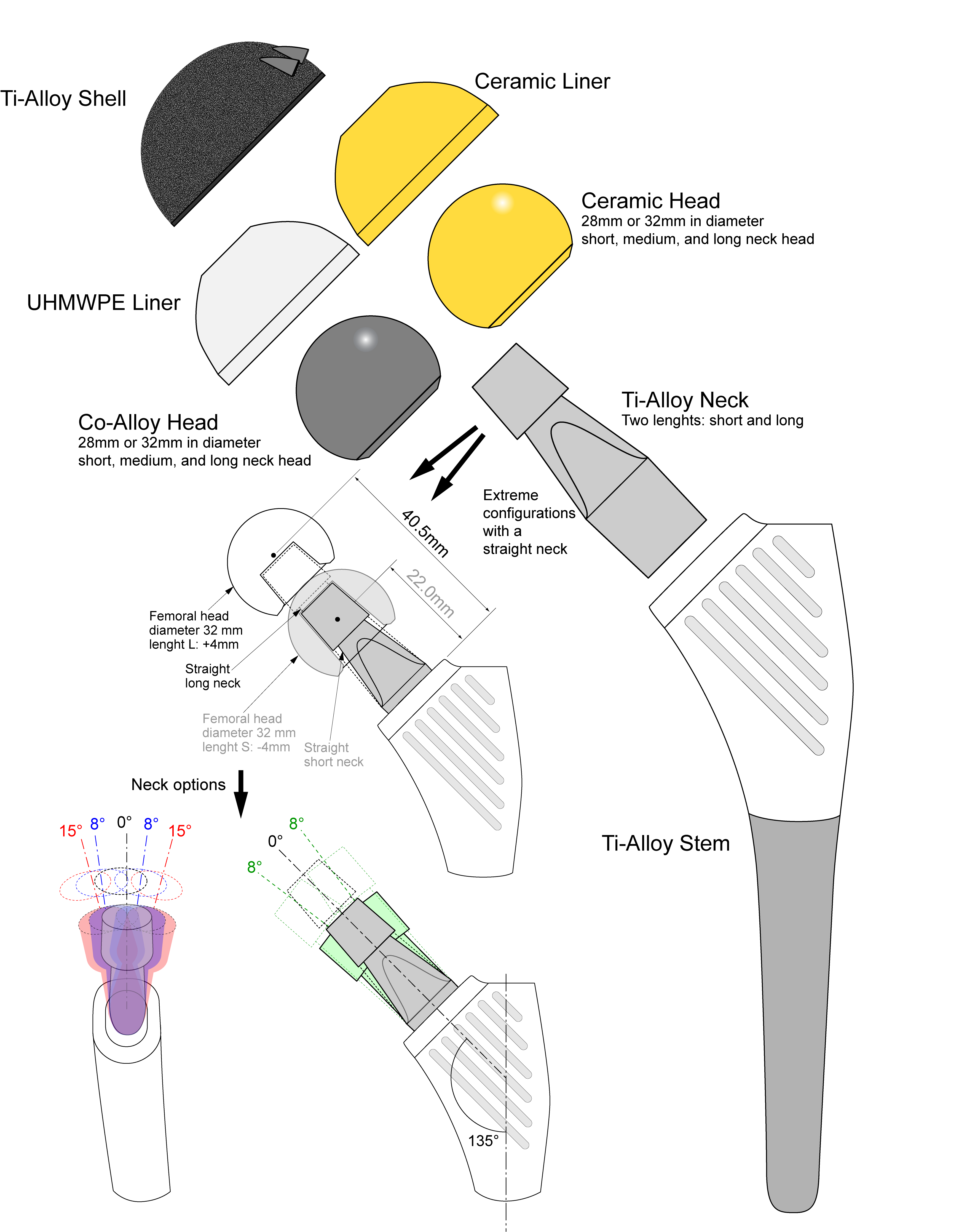

Supplement: Supplementary file 3 — Supplementary file3 The AncaFit acetabular component was a Ti-alloy (Ti6Al4V) porous acetabular shell assembled with a polyethylene or ceramic liner. The femoral component was an anatomically shaped stem, made of Ti-alloy (Ti6Al4V), with an exchangeable-neck, made of Ti-alloy (Ti6Al4V). The exchangeable-neck stem had a proximal 12/14 taper allowing the assembly of a Co-alloy or ceramic head. Two head diameters (28 mm, 32 mm) and three head lengths (S, M, L) were available in the inventory. Additionally, two neck lengths (short and long neck) were available. Therefore, the distance of the head center with respect to the neck–stem engagement level ranged from 22.0 mm to 40.5 mm. Six neck versions were available: straight, 8° and 15° angled neck in antero/retro direction, 8° angled neck in varus/valgus, and two hybrid neck designs combining 4° angle in varus/valgus and 6° angle in antero/retroversion (not shown in the scheme to make the figure clearer). Note, the 15° angled neck in varus/valgus was not available at that time (TIF 45916 kb) [file 402_2022_4634_MOESM3_ESM.tif]
